# ABC’S of AI: proposing a clinician’s framework for the interpretation of artificial intelligence studies

**DOI:** 10.3389/fmed.2026.1787075

**Published:** 2026-03-11

**Authors:** Brian Murray, Seth R. Bauer, Yanjun Gao, Erin F. Barreto, Kelli Henry, Kaitlin Blotske, Andrea Sikora

**Affiliations:** 1 University of Colorado Skaggs School of Pharmacy, Aurora, CO, United States; 2 Department of Pharmacy, Cleveland Clinic, Cleveland, OH, United States; 3 University of Colorado School of Medicine, Aurora, CO, United States; 4 Mayo Clinic, Rochester, MN, United States

**Keywords:** analysis, artificial intelligence, critical care, framework, machine learning

## Abstract

Artificial intelligence (AI) and machine learning have emerged as transformative analytic methods capable of identifying patterns and proposing treatment strategies from complex, heterogeneous data generated in patient care settings. However, translating reports of AI tools into clinical decision-making requires careful interpretation and contextualization. Here, we present a structured framework (ABCDEFG framework) for clinicians to critically appraise studies reporting AI tools using a high-profile reinforcement learning analysis of vasopressin initiation in septic shock as a case example. We highlight key methodological strengths including adherence to reporting standards, alignment with clinical outcomes, and external validation. We also identify limitations related to causal inference, reward structure design, assumptions about state representation, and implementation readiness. Our framework emphasizes the importance of rigorous, clinically grounded critique before integrating AI tools into practice, and provides a roadmap for clinicians to assess the reliability and relevance of emerging AI-based decision support.

## Introduction

The ability to critique and interpret results from studies reporting artificial intelligence (AI) and machine learning tools is becoming essential for clinicians. Despite impressive feats like diagnostic performance on complex medical cases (1) and early warning systems that predict clinical deterioration hours before standard of practice (2), AI technologies are not a panacea for clinical practice and require thoughtful analysis before adoption decisions.

**Figure 1:**
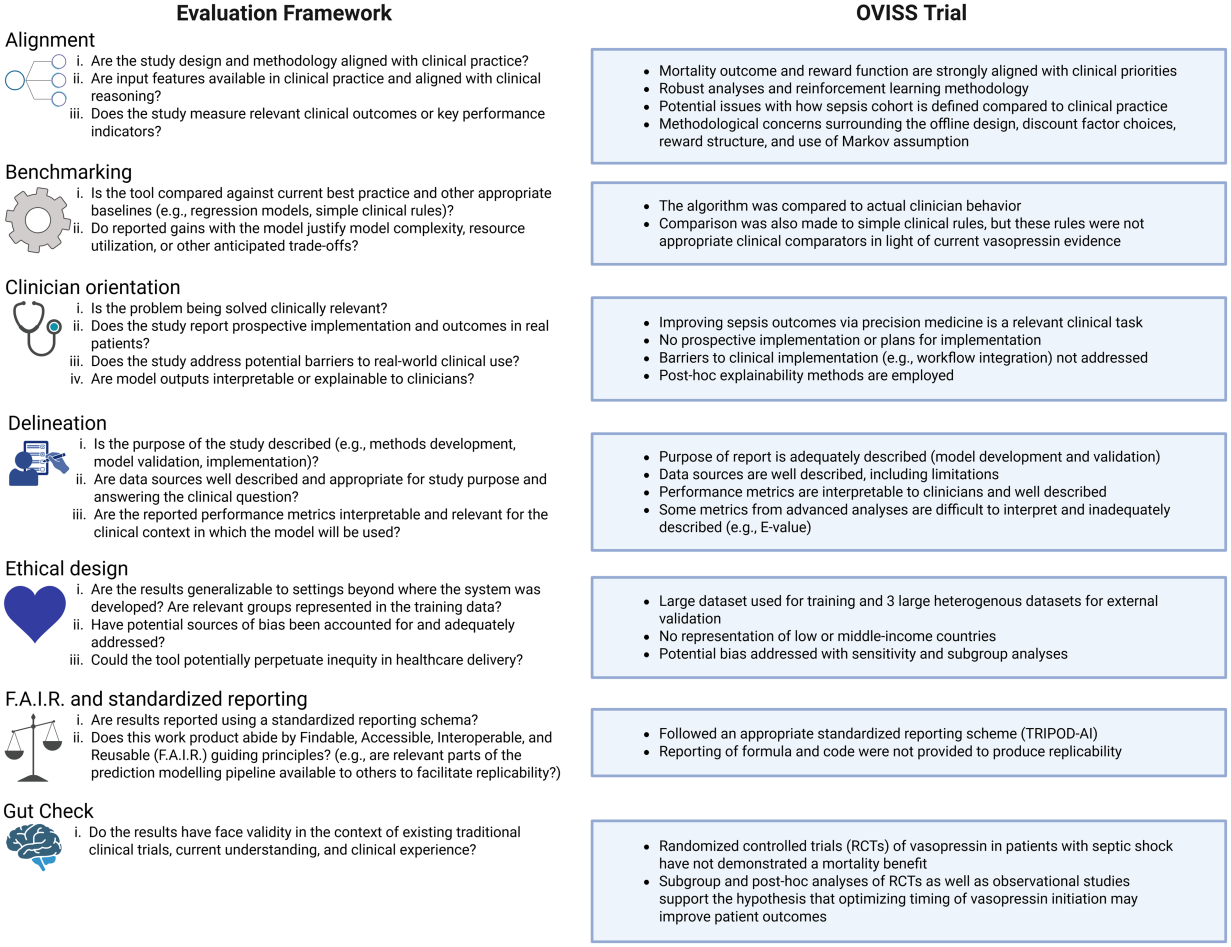
An illustrative journal club for best practices in the peer-review and critical analysis of AI-based evaluations. Created with BioRender.com.

For many clinicians, analyzing reports of AI tools will at first be unfamiliar. We propose the Alignment, Benchmarking, Clinician orientation, Delineation, Ethical design, F.A.I.R. and standardized reporting, and Gut check (ABCDEFG) framework to guide clinicians when interpreting the performance and outcomes associated with AI tools (Figure 1). We contextualize the framework using the Optimal Vasopressin Initiation in Septic Shock (OVISS) study as an example. Importantly, discussion of OVISS within this framework is not intended to adjudicate methodological choices, but to illustrate how clinician understanding of limitations and trade-offs is critical for contextualizing results and informing translation into practice.

## Brief study overview

In 2025, Kalitmouttou et al. published the OVISS study evaluating a *reinforcement learning* model trained to identify the optimal timing for initiation of vasopressin following the onset of septic shock (3). The study included more than 14,000 patients and projected an impressive mortality reduction if the model had guided vasopressin initiation [odds ratio (OR), 0.81; 95% confidence interval (CI), 0.73–0.91]. Table 1 presents key terminology.

**Table 1:** Terminology.

Terminology	Definition
Causal inference	A set of methods used to determine whether a treatment or action actually causes a change in an outcome, rather than simply being associated with it.
Counterfactual	An outcome that represents what would have happened if a different decision had been made.
Discount factor	A modifiable parameter that changes how a model preferences immediate versus future rewards. Discount factors closer to 1 prioritize long-term rewards (e.g., mortality), while discount factors closer to 0 prioritize short-term rewards (e.g., physiological improvement).
eICU-collaborative research database (CRD)	Large multicenter critical care database including over 200,000 ICU admissions from over 200 institutions in the years 2014 and 2015.
E-value	A statistical measure of how sensitive an observed association is to unmeasured confounding. For example, an E-value of 1.46 indicates that an unmeasured confounder associated with both vasopressin exposure and the measured outcome by at least 46% each could fully explain the observed association.
Markov assumption	The assumption that a patient’s future condition depends only on their current state as defined by a set of variables and on the action taken, not on historical information about how the patient arrived in that state.
MIMIC-IV	A large de-identified dataset of patients admitted to the emergency department or an ICU at Beth Israel Deaconess Medical Center, with the most recent version including over 300,000 unique individuals and nearly 550,000 hospital admissions from 2008–2022.
Off-line models (static models)	Machine learning models that are trained with a fixed retrospective dataset. These models do not update their decisions while caring for real patients and cannot test different treatment options in real time.
Off-policy evaluation	A method used in reinforcement learning in which a model’s recommendations are applied to historical patient data, and outcomes observed under real-world clinician decisions are compared with the outcomes the model predicts would have occurred if its recommendation had been followed.
Overfitting	When a model is too closely tailored to the data it was trained on, causing it to work well on the training dataset but perform poorly on new patients because it learned patterns that do not generalize.
Reinforcement learning	A type of unsupervised machine learning where rewards and penalties are given as a part of feedback to improve the model’s ability to make decisions.
Shapley additive explanations (SHAP)	A method that explains model decisions by assigning each input variable a numeric value showing how much that variable influenced the model’s recommendation, helping clinicians understand the reason behind the model’s output.
State aliasing	When a model cannot distinguish between different situations and applies the same actions, even if a situation warranted a different response from the model.

## Applying the ABCDEFG framework

### Alignment with clinical practice

Before evaluating technical performance, clinicians should assess whether an AI tool is aligned with clinical reasoning and priorities. This includes examining whether the model uses clinically meaningful features and optimizes outcomes that are patient-centered. Models that align poorly with clinical constructs may perform well but be difficult to apply in practice (4).

OVISS explores mortality, a patient-centered and clinically relevant outcome. The reward structure also heavily prioritized long-term outcomes (*discount factor* = 0.99) and assigned a substantial penalty to mortality (−20), aligning with clinical incentives. However, these modeling choices introduce trade-offs. High discount factors risk amplifying noise and producing a policy that lacks generalizability (5, 6). Although OVISS performed sensitivity analyses with discount factors ranging from 0.90–0.99, all emphasize long-term rewards heavily. Additional sensitivity analyses using lower discount factors (e.g., 0.5–0.7) could help distinguish near-term treatment effects from long-term reward optimization.

Similarly, while the reward structure (e.g., −20 for death, +1 for norepinephrine dose decrease) appears clinically intuitive, it lacks empirical calibration. Additionally, repeated small surrogate rewards (e.g., vasopressor reduction) can accumulate across epochs and disproportionately influence the policy, potentially outweighing the penalty assigned to mortality. A sensitivity analysis was performed using equal weighting for each reward component, but this approach further diluted mortality importance. Performing a mortality-dominant sensitivity analysis clarifies whether projected benefits are maintained when surrogate rewards cannot exceed the mortality penalty (7).

OVISS also relied on the *Markov assumption*, modeling decisions from a limited set of 14 current-state variables. This approach simplifies modeling but increases the risk of s*tate aliasing* by neglecting clinically relevant information (e.g., trends, prior therapeutic decisions, confounders) that influence both current state and future outcomes, especially in complex diseases like sepsis. Augmenting state representation with recent trends, treatment history, and a more robust set of baseline and current-state variables with special attention to key confounders and decision drivers (e.g., timely and appropriate antibiotics) increases model complexity but also reduces risk of state aliasing and improves alignment with clinical decision making.

Finally, case identification is a fundamental challenge facing retrospective studies of sepsis and septic shock. While Sepsis-3 clinical criteria were used in OVISS, a detailed description of cohort identification is warranted to improve external validity, especially considering characteristics of two of the major retrospective datasets used to validate the model (i.e., *MIMIC-IV* and *eICU-CRD*) (8, 9) preclude reliable identification of patients with septic shock via Sepsis-3 criteria. Even subtle variations in sepsis cohort identification (e.g., defining sepsis onset time with transposed suspicion of infection and organ dysfunction criteria times) have led to striking differences in characteristics and outcomes that can impact model performance (10, 11).

### Benchmarking against relevant clinical standards

AI models must be compared against the care approaches they aim to improve. Benchmarking against relevant comparators, including current state and simpler rules or models (e.g., comparing performance of a mortality prediction model to standard severity of illness scores), better delineates the value added by complex methods. Without appropriate comparators, it is difficult to determine whether a model’s performance reflects true clinical insight.

The OVISS study compared patient outcomes projected under the model’s learned policy against those observed under clinician-guided care representative of current clinical practice. However, OVISS did not compare the AI model to a simpler model, which could help determine whether performance stems from true learning or favorable reward shaping. Simple clinical rules for vasopressin initiation (i.e., serum lactate > 4 mmol/L, norepinephrine dose > 0.7 mcg/kg/min, MAP < 65 mmHg 12 h after shock onset) were used as comparators. Problematically, the rules chosen are not clinically relevant. Current Surviving Sepsis guidelines and evidence suggest benefit with earlier initiation of vasopressin; more apt simple clinical rules for comparison could be lactate > 2 mmol/L, norepinephrine dose > 0.25 mcg/kg/min, or 6 h from shock onset (12–14).

### Clinician orientation

AI tools must be trusted and accepted by clinicians before use in clinical practice. Even well-performing models may not translate into practice if they are difficult to understand or conflict with clinical intuition. Though efforts were made to support clinician orientation, there remained a significant gap between model policy and clinician behavior in OVISS.

Several features in the OVISS trial are notable for incorporating strategies that support clinician orientation. For one, the model was trained in a large, diverse cohort and externally validated in three independent datasets (i.e., MIMIC-IV, eICU-CRD, and a separate health system dataset), strengthening generalizability and reducing potential for *overfitting*. Sensitivity and robustness analyses were also performed to evaluate the impact of reward structure, modeling assumptions, and potential confounding. While individual design choices within these analyses may be open to critique, the breadth of sensitivity testing is commendable and provides insight into how analytic decisions influenced model behavior. To improve explainability and address the “black box” nature of complex AI tools, post-hoc *Shapely Additive Explanations (SHAP)* were used to identify variables most influential to model decisions.

The *off-line* model design, while appropriate for development and validation, limits clinician assessment of real-world applicability. Because the model learns only from retrospective clinician decisions, it cannot observe *counterfactuals* or interactively test its policies in a real-time environment, limiting *causal inference* unless stringent assumptions are met. Important confounders influencing treatment and outcomes (e.g., empiric antibiotics, bacterial culture results, successful control of the infectious source, use of other vasopressors) were not captured, and retrospective datasets may lack information on additional relevant confounders. Moderate robustness to confounding (*E-value* of 1.46) suggests that relatively small unobserved differences could influence results.

### Delineated purpose and methods

Implementation studies can be considered the Phase 3 clinical trial of AI tools (15); without them it is impossible to assess critical factors beyond base model performance that determine safety and effectiveness (e.g., clinical workflow integration, algorithm aversion). While not all tools are ready for clinical implementation, all phases of development are relevant. As such, it is critical that reports of AI tools clearly define the purpose of the study.

Like most AI tools in critical care (16, 17), OVISS does not report prospective implementation. Instead, model evaluation used *off-policy evaluation (OPE)* to estimate outcomes under model-recommended actions compared with observed clinician behavior. While OPE is a necessary and appropriate framework for hypothesis-generating studies, inherent methodological limitations prevent it from replacing prospective model implementation for the assessment of safety and efficacy. Importantly, the authors acknowledge this distinction by clearly defining their purpose to be derivation and validation of a decision policy while providing a clear use case for future application.

### Ethical design

Equitable and responsible AI requires explicit attention to bias, fairness, and differential performance across patient groups. Because models learn patterns from historical data, they can reproduce or amplify inequities unless safeguards are built into model design and evaluation. In addition to external validation, OVISS executed appropriate subgroup and sensitivity analyses to address risk of bias, fairness, and equity. While many additional ethical considerations are central to responsible AI implementation, a comprehensive ethical review is beyond the scope of this work. Several high-quality frameworks and consensus guidelines addressing these issues are available elsewhere (18–22).

### F.A.I.R. and standardized reporting

Transparent reporting enables clinicians to evaluate whether a model was developed rigorously and can be reproduced in other settings. Similar to demographics tables or intervention descriptions in traditional clinical trials, necessary and sufficient information for appraisal must be reported for AI tools (23). Reporting frameworks for various study types evaluating AI tools have been developed and are available through the Equator network (24–33). The OVISS study appropriately adheres to and provides a checklist of framework elements for the Transparent Reporting of a multivariable prediction model for Individual Prognosis or Diagnosis (TRIPOD)-AI reporting framework, which is used for reporting clinical prediction models including those utilizing machine learning methods.

### ‘Gut check’ (clinical plausibility)

A key consideration is whether results are clinically plausible in the context of current understanding, clinical experience, and traditional studies evaluating the intervention. In the case of vasopressin, use and management in septic shock is highly variable (34). Guidelines suggest initiation of vasopressin for patients with septic shock on norepinephrine with inadequate mean arterial pressure, noting some initiate vasopressin when norepinephrine dose reaches 0.25–0.5 mcg/kg/min (12). Randomized controlled trials and meta-analyses of vasopressin analogues in septic shock demonstrate improved secondary outcomes (e.g., catecholamine dose requirements) but only low-certainty evidence of a mortality advantage [relative risk (RR) 0.95; 95% CI, 0.87–1.04] (35–38). Considering this context, OVISS demonstrated a mortality benefit above expectations, warranting caution. However, subgroup and post-hoc analyses of the Vasopressin and Septic Shock Trial (VASST) study and several observational studies support the hypothesis that patient selection and timing of initiation may be critical factors determining the impact of vasopressin on mortality (13, 38, 39). Though lower-level evidence, these findings suggest a model optimizing vasopressin initiation may improve outcomes.

## Integrating the framework

Applying the ABCDEFG framework to OVISS illustrates how AI studies may present compelling and clinically relevant findings while still warranting careful contextual interpretation. By evaluating these key domains, clinicians can determine where an AI tool falls on the spectrum from hypothesis-generating analysis to implementation readiness. This structured appraisal supports balanced decision-making: recognizing promise and innovation while remaining grounded in evidence-based practice.

## Conclusion

As AI becomes increasingly integrated into clinical research and practice, the ability of clinicians to appraise these tools will be essential to their safe, effective, and equitable application. Using the proposed standardized framework, clinicians can confidently and critically evaluate AI literature and guide integration into clinical practice.

Following a structured evaluation framework enables clinicians to confidently critique and appraise methodology, strengths, limitations, and ultimately value and clinical applicability of new AI tools. The proposed ABCDEFG framework (Alignment, Benchmarking, Clinician orientation, Delineation, Ethical design, F.A.I.R. and standardized reporting, and Gut check) covers domains essential to the clinician’s assessment of AI tools. This framework is proposed as a practical scaffold for bedside clinicians evaluating reports of AI tools.

## Data Availability

The original contributions presented in the study are included in the article/supplementary material, further inquiries can be directed to the corresponding author.
